# An Old World leaf‐cutting, fungus‐growing ant: A case of convergent evolution

**DOI:** 10.1002/ece3.9904

**Published:** 2023-03-15

**Authors:** A. Dejean, P. Naskrecki, C. Faucher, F. Azémar, M. Tindo, S. Manzi, H. Gryta

**Affiliations:** ^1^ Laboratoire écologie fonctionnelle et environnement Université de Toulouse, CNRS, Toulouse INP, Université Toulouse 3 – Paul Sabatier (UPS) Toulouse France; ^2^ UMR EcoFoG, AgroParisTech, Cirad, CNRS, INRA Université des Antilles, Université de Guyane Kourou France; ^3^ Museum of Comparative Zoology Harvard University Cambridge Massachusetts USA; ^4^ Laboratoire Evolution & Diversité Biologique, Université de Toulouse, CNRS, IRD Université Toulouse 3 ‐ Paul Sabatier, 118 route de Narbonne Toulouse France; ^5^ Laboratory of Animal Biology and Physiology, Faculty of Science University of Douala Douala Cameroon; ^6^ Present address: Institut de Pharmacologie et de Biologie Structurale (IPBS), CNRS Université Toulouse Toulouse Cedex France

**Keywords:** ant–fungus relationships, carton nest structure, *Crematogaster*, defoliation, evolution, mycelium‐composite material

## Abstract

The African myrmicine ant *Crematogaster clariventris* is a territorially dominant arboreal species that constructs very hard carton nests. Noting that workers cut off leaves from different plant species while building or repairing their nests, we asked ourselves if there was a correlation. We conducted scanning electron microscopic observations of nest walls that revealed the presence of fungal mycelia. As the presence of filamentous Ascomycota has been shown on arboreal ant nests worldwide, we used a metabarcoding approach and, indeed, noted the presence of Operational Taxonomic Unit (OTU) Cre_006041 of the Capnodiales known to reinforce large nests of an unidentified African *Crematogaster*. This OTU was also recorded in the workers' bodies. At a very low level, we also noted OTU Cre_320021 of the Chaetothyriales known for their relationships with the African plant‐ant species *C*. *margaritae*. Therefore, by cutting leaves and growing fungus, *C*. *clariventris* illustrates a case of convergent evolution with higher New World leaf‐cutting, fungus‐growing Attina of the genera *Acromyrmex*, *Amoimyrmex* and *Atta*. However, there are notable differences. Leaf‐cutting Attina cultivate Agaricaceae (Basidiomycota) for food, whereas *C*. *clariventris* uses Capnodiales to reinforce their nests (i.e., after the mycelium died, the hyphae's cell walls remained sturdy forming a natural composite material), have a distinct geographical origin (i.e., New World vs. Old World) and belong to a distinct ant tribe in the subfamily Myrmicinae (i.e., Attini vs. Crematogastrini). Furthermore, leaf‐cutting Attina evolved an efficacious means of cutting leaves by using their mandibles asymmetrically, whereas *C*. *clariventris* workers, typically, use their mandibles symmetrically.

## Graphical abstract

*Crematogaster clariventris*, a myrmicine ant from the African subgenus Atopogyne, is a defoliator that uses vegetal material to grow fungi as do higher New World leaf‐cutting Attina, illustrating a case of convergent evolution. However, *C*. *clariventris* grows fungal mycelia to reinforce the carton of the nests rather than cultivating it for food.

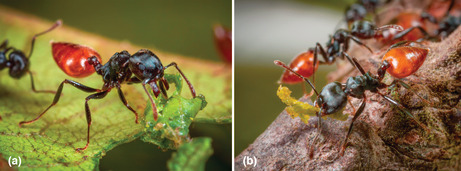

## 1 INTRODUCTION

The massive amounts of dead plant biomass following the Cretaceous–Paleogene (≈65.5 Mya) mass extinction event resulted in a proliferation of saprophytic fungi. Overtime, they developed different types of interactions with all other living terrestrial organisms, including ants that appeared during the Early Cretaceous and diversified alongside the rise of angiosperms with which they evolved diverse relationships, from defoliation to symbiotic mutualisms (Heitman et al., 2018; Maruoka, 2019; Moreau et al., 2006).

The most notable ant–fungus mutualism is the obligate food‐based symbiosis involving the “Attina” New World subtribe (subfamily Myrmicinae; tribe Attini) that appeared at the end of the Paleocene between 61 and 57 Mya during the recovery period that followed the second mass extinction. These ants cultivate Basidiomycota Agaricaceae (exceptionally, *Apterostigma* species in the *pilosum* group cultivate Pterulaceae) in fungal gardens from which they remove parasitic fungi or eliminate them thanks to the antifungal actinomycete bacteria growing on their cuticle. A facultative mutualism evolved with the lower Attina that domesticated cultivars from free‐living populations they provision with dead insects, feces and fallen vegetal debris. In an obligate mutualism, the higher Attina cultivate derived, specialized clades incapable of surviving on their own that produce lipid‐ and carbohydrate‐rich hyphal tip swellings, the gongylidia, specifically to feed these ants. Furthermore, an ecological innovation appeared 18–19 Mya (Miocene) with leaf‐cutting behavior among the higher Attina of the genera *Acromyrmex*, *Amoimyrmex* and *Atta* that supplied the fungal cultivar with fresh plant parts, mostly leaves (Barrera et al., 2022; Branstetter et al., 2017; Cristiano et al., 2020; Mueller et al., 2018; Sapountzis et al., 2019; Schultz, 2021).

Other cases of food‐based ant–fungus mutualisms have been noted for some “plant‐ants” associated with myrmecophytes (i.e., plants sheltering colonies of some ant species in hollow structures called domatia), as they feed on the thin‐walled Chaetothyriales (Ascomycota) that line the domatia (Blatrix et al., 2012; Greenfield et al., 2021; Mayer et al., 2018; Vasse et al., 2017). Also, some thin‐walled Chaetothyriales produce antibiotics that protect arboreal ant nests from pathogens (Lucas et al., 2019; Moreno et al., 2019).

Finally, another ant–fungi mutualism is seen in filamentous Ascomycota of the orders Capnodiales and Chaetothyriales used to reinforce the carton of the nests and/or galleries of different ant species. This carton is composed of chewed trichomes and other plant tissues into which the ants introduce these fungi. The hyphae consist of tubular cells whose walls are mainly composed of chitinous microfibers, glucans and glycoproteins. During the hyphae's apical growth, the tube‐shaped cell walls trail behind, persist and form tubular, highly resistant fibers. Keeping their structural properties, they remain sturdy after the death of the mycelium, reinforcing the ants' constructions (Baker et al., 2017; Dejean et al., 2005; Heitman et al., 2018; Jones et al., 2020; Quan et al., 2021, 2022; Ruiz‐González et al., 2011; Schlick‐Steiner et al., 2008; Vasse et al., 2017; Voglmayr et al., 2011).

**FIGURE 1:**
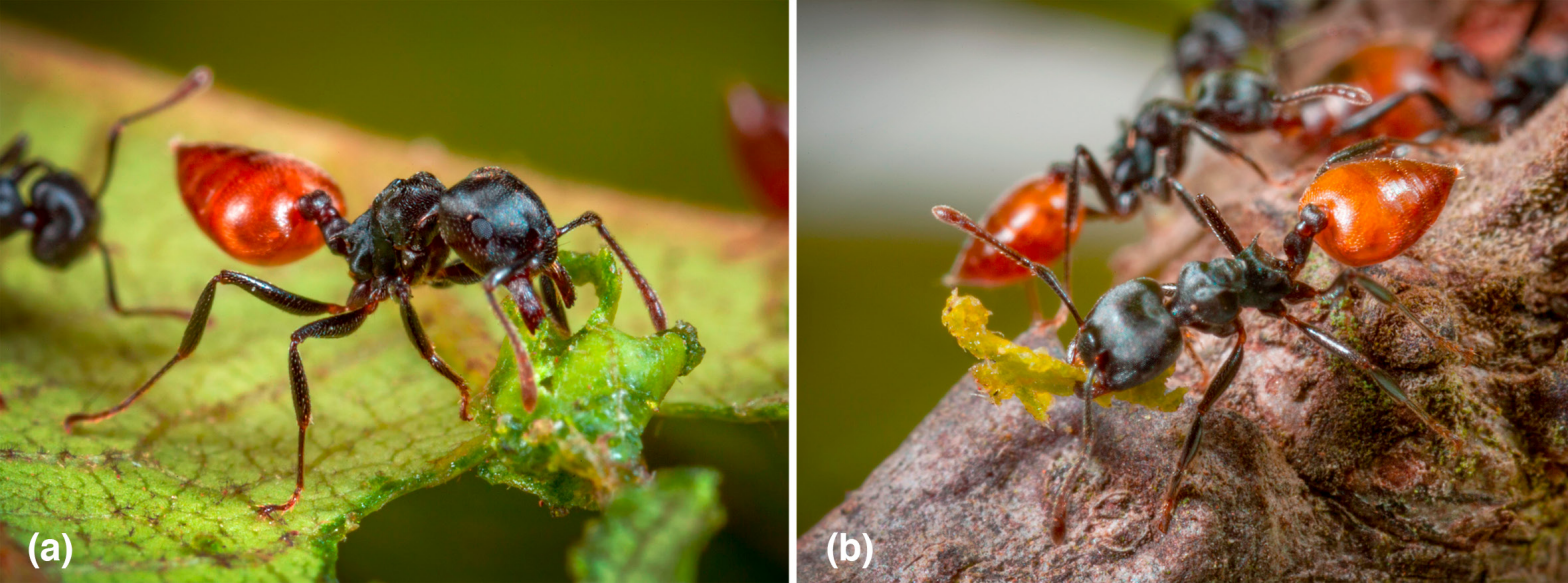
*Crematogaster clariventris* cutting (a) or retrieving (b) pieces of young nitrogen‐rich leaves. The characteristic yellow gaster of the workers is not always as striking as in this figure (Photos Piotr Naskrecki).

Because we noted that the workers of the African *Crematogaster clariventris* were cutting leaves (Figure 1) and transporting small pieces of vegetal material while building new arboreal carton nests, we hypothesized that these two activities were correlated if this species effectively also grows fungi. Thus, we aimed (1) to clarify if *C*. *clariventris* truly cuts leaves to build or repair their nests, (2) to ascertain through scanning electron microscopic observations if fungal hyphae are components of the nests' carton structure, (3) to determine the fungal community present in the nests using a metabarcoding approach, and (4) to identify the fungal candidates grown by *C*. *clariventris* by comparing them with well‐known species previously recorded worldwide.

## 2 MATERIALS AND METHODS

### 2.1 Study sites and focal species

Observations, field studies and sampling were conducted in Cameroon on trees separated by several kilometers (Yaoundé: 3°51′N; 11°29′E; Nkolbisson: 03°59′N, 11°28′E; Kala: 03°50′N, 11°21′E).

*Crematogaster clariventris* is a myrmicine ant belonging to the African monophyletic subgenus *Atopogyne* (tribe Crematogastrini, “Global *Crematogaster*” clade) that, like higher leaf‐cutting Attina, appeared ≈15 Mya (Miocene: 23.03–5.33 Mya; Blaimer, 2012). Populous colonies of this territorially dominant arboreal species build several large, very hard carton nests on the main branches of canopy trees (30–45 m in height) (Dejean et al., 2016). The polymorphic workers (≈4.5 mm in length for the majors) are characterized by a yellowish gaster. Voucher specimens were deposited in the Museum of Natural History, London.

### 2.2 Field observations and experiments

First, we studied the rhythm of activity of *C*. *clariventris* workers defoliating relatively small (up to 4 m in height) plants, identified the species they selected and noted what parts they attacked. Using a magnifying glass, we also observed the workers cutting pieces of vegetal material to compare this process with that of leaf‐cutting Attina. Second, using a machete, we opened *C*. *clariventris* nests found on recently downed trees, permitting us to observe their internal structure. Third, to test if the defoliation is directly related to nest construction, we conducted a pre‐post analysis where we compared the day before and the day after we removed a ≈ 1 dm^3^ piece of the largest accessible nest from each of three geographically distant colonies at ≈12:00 (the activity needed for typical maintenance of the *C*. *clariventris* polydomous nests with the activity required to repair the damaged nest). At 10:00 a.m., 11:00 a.m., 12:00 p.m., 01:00 p.m. and 02:00 p.m., we conducted 5‐minute sessions of direct observations of the number of workers transporting a piece of clearly visible vegetal material from zones selected for their relative proximity to the main nests. This experiment was conducted 2 months later on the same colonies, but the ≈1 dm^3^ piece of nest was removed from a new, slightly smaller nest. The values obtained (i.e., a total of 30 cases for “before and after” the removal of parts of the nests) were analyzed using a GLM‐Poisson regression (Wald test). The response variable was the number of workers transporting vegetal material the covariates were the colony and the hour interacting with the treatment the “day before or the day after” (R software).

### 2.3 Looking for fungal mycelia in the carton of the nests

Parts from three *C*. *clariventris* nests (≈0.5 dm^3^) were collected and transported to the laboratory and then frozen. Pieces of intact inner nest walls were observed using a scanning electron microscope (Hitachi TM‐1000 Tabletop).

### 2.4 Metabarcoding nest fungi and screening for known associated fungi

We characterized the fungal community of the intact and repaired parts of nest walls using a Next Generation DNA Sequencing/metabarcoding approach. Samples (CN1, CN2 and CN3) of ≈80 mg were taken and conserved at −20°C in Nuclei Lysis Solution (Promega) until DNA extraction. A tube containing only Nuclei Lysis Solution served as a control throughout all the procedures. Thawed samples were ground with a pestle, and total DNA was extracted and purified using the WIZARD Genomic DNA Purification Kit (Promega) according to the procedure developed by Carriconde et al. (2008). Sample DNA concentrations were evaluated with a Qubit 2.0 fluorometer (Invitrogen‐Life Technologies) and normalized to 5 ng/μL.

The fungal internal transcribed region 1 of nuclear ribosomal DNA (ITS1) was amplified with ITS5 forward primer (GGAAGTAAAAGTCGTAACAAGG; White et al., 1990) and a modified version of 5.8S_Fungi reverse primer (CAAGAGATCCGTTGTTGAAAGTK; Epp et al., 2012). Forward and reverse primers were synthetized with a combination of two different 8‐nucleotide tags per sample to discriminate samples after sequencing. As such, each DNA extract was amplified with a unique combination of tagged primers as in Nagati et al. (2018). The amplified products were pooled, and the library was prepared with the Illumina TruSeq Nano PCR‐free kit (Illumina) before sequencing by the GENETOUL GeT‐PlaGe core facility (Toulouse) on an Illumina MiSeq platform by using the paired‐end sequencing technology (2 × 250 pb) with the MiSeq Reagent Kit v3.

The OBITools package (Boyer et al., 2016) was used to analyze the raw data obtained from the Illumina sequencing. Then, after a paired‐end read assembly, read assignment to samples and read dereplication, we discarded the sequences with ambiguous nucleotides or a low pair‐end alignments score, as well as those shorter than 60 pb. A data matrix of sequence (reads) abundance across samples was obtained. Sequences were clustered with a threshold of 97% similarity using OBITool Sumaclust, and each cluster was considered an OTU (see 3‐Tab in Dejean et al., 2022). OTUs represented by less than 15 reads and OTUs with more reads in the control sample than in one of the three experimental samples were not considered.

The closest matches to the most abundant sequence for each OTU were obtained from the GenBank database (http://www.ncbi.nlm.nih.gov/) by following a BLAST procedure (Altschul et al., 1990). A taxonomic rank was then assigned to each OTU according to the location of its sequence in a Neighbor Joining tree constructed with the 50 closest GenBank sequences (NCBI tree widget). The traits of fungi having the closest sequences were examined in order to detect the fungal OTUs that potentially interact with ants and thus may be involved in nest construction.

As only members of Capnodiales *s*.*l*. and of Chaetothyriales were isolated from hyphae with a structural function in ant cartons, whereas the other strains, mostly coming from spores, are most likely contaminants (Vasse et al., 2017), we investigated the phylogenetic locations of the recorded OTUs for these two orders. For each of them, a dataset including ITS sequences of known ant‐associated fungi and of fungi representative of the families of these two orders was constituted and used to construct maximum likelihood phylogenies for these two orders (see Tables S1 and S2 in Dejean et al., 2022). The sequences of the dataset and the ITS1 sequences of the OTUs were first aligned with MUSCLE v3.7 (Edgar, 2004) implemented in GENEIOUS 6.1.8 (https://www.geneious.com), and the alignments were manually refined. Phylogenetic analyses were conducted with PHYML 3.0 (Guindon & Gascuel, 2003) using a GTR + gamma evolutionary model with 100 bootstraps, and trees were edited with MEGA7 (Kumar et al., 2016).

### 2.5 Specific PCR‐amplification of rDNA ITS of the OTU of interest in ants' bodies and in nests

*Crematogaster clariventris* workers were sampled (CW1, CW2 and CW3) from nests, and the presence of one OTU of interest selected through metabarcoding (i.e., OTU Cre_006041) was searched for in workers' bodies through a specific PCR approach. Fungi are typically carried by ants in the infrabuccal pocket or, as noted for plant‐ants by Pringle and Moreau (2017), on the gaster. So, in order to eliminate fungal spores, hyphae and other fragments stuck on the workers' exoskeletons prior to searching for the presence of fungi inside their bodies, each ant was surface‐sterilized with 70% ethanol for 1 min and with 10% sodium hypochlorite for 1 min and rinsed in sterile water. DNA was further extracted from workers as described above for the nests.

In order to specifically detect the OTU Cre_006041 by direct PCR in the ants' bodies and in nest samples, a forward PCR primer, CreCapnoF (5′ CTCCAACCCTTTGTCTCCAA 3′), located in the ITS1 region of rDNA and specific to ITS1 sequences of “Carton Clade 3” (Voglmayr et al., 2011) to which the OTU Cre_006041 belongs, was designed. The CreCapnoF primer was then used with ITS4 primer (White et al., 1990) to amplify a rDNA region including part of ITS1, 5.8S and ITS2 in DNA extracted from ant and nest samples. PCR reactions were carried out in a final volume of 25 μL containing 1X of GoTaq green buffer (Promega), 0.2 mM of each dNTP, 1 μM of each primer, 1 U of GoTaq G2 Hot Start polymerase (Promega) and 2 μL of DNA extract, with PCR cycling conditions as follows: initial denaturation at 94°C for 3 min; 35 cycles at 94°C for 45 s, 53°C for 45 s, 72°C for 1 min; and a final extension at 72°C for 10 min. A negative control without DNA was included in the PCR run. Amplified fragments were UV visualized after electrophoresis in a 0.5× TAE buffer on 1.4% agarose gel including ClearSightDNA (Euromedex).

To ensure the proper identification of the PCR products obtained, they were sequenced by EUROFINS (Ebersberg) in both directions with the CreCapnoF and ITS4 primers, and the sequences were compared to the OTU Cre_006041 sequence obtained through metabarcoding and to the ITS sequences of fungi belonging to “Carton Clade 3” (Voglmayr et al., 2011).

## 3 RESULTS

### 3.1 Field observations and experiments

**FIGURE 2:**
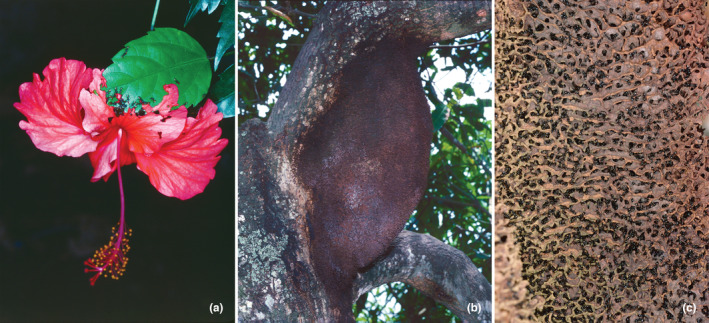
*Crematogaster clariventris* in the process of cutting pieces of a nitrogen‐rich *Hibiscus rosa‐sinensis* (Malvaceae) flower (a). They also defoliate *Albizia* spp. (Fabaceae), *Alchornea cordifolia* (Euphorbiaceae), *Cassia* spp. (Fabaceae), *Mangifera indica* (Anacardiaceae) and *Theobroma cacao* (Malvaceae). A *Crematogaster clariventris* carton nest (b) and details of the carton inside the nest (c) (photos Alain Dejean).

**FIGURE 3:**
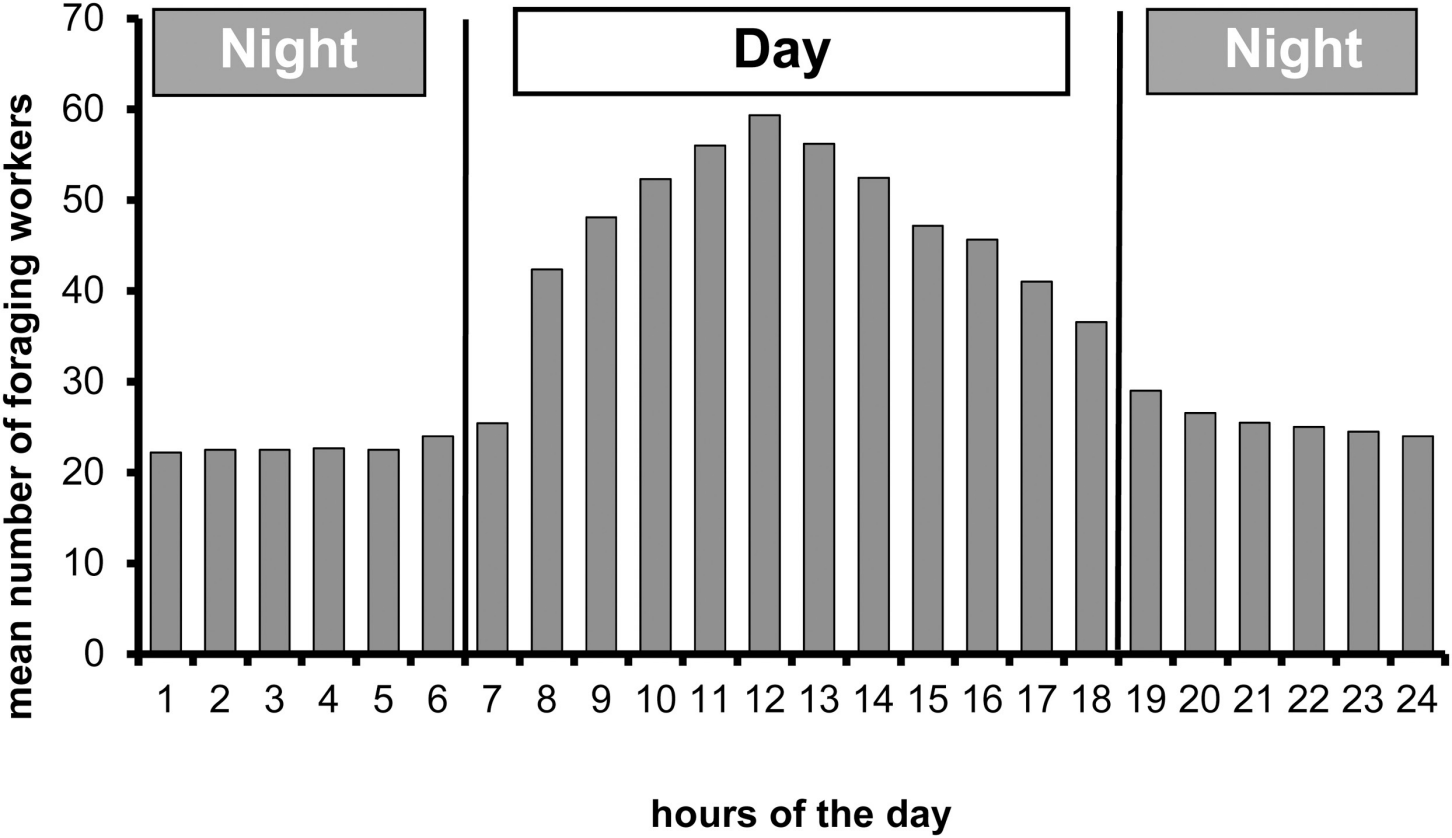
Rhythm of activity of *Crematogaster clariventris*. Number of individuals retrieving clearly visible pieces of vegetal material. This study was conducted on three colonies. Selecting a branch serving as a bridge providing access to the host trees so that the workers were seen in profile, we counted during 5 min per hour and per colony the number of workers retrieving a clearly visible piece of vegetal material. Six to 12 replicates were conducted each hour of the nychthemeron during 8 days of observations.

Defoliating *C*. *clariventris* workers selected the nitrogen‐rich young leaves or flowers (see Kursar & Coley, 1991; Mattson Jr., 1980) of different plant species from which they cut out relatively small pieces of very different shapes of up to 4 mm in length (Figures 1 and 2a). The defoliating activity, observed all around the clock, was higher during the daytime, particularly between 8:00 a.m. and 02:00 p.m. (Figure 3).

The experimental removal of a part of the nests from three geographically distant colonies was followed by a significantly higher number of workers retrieving a piece of vegetal material the “day after” (Wald test: *p* = .02; the covariates were not significant). Among the workers that repaired the nest damage, foragers transported a clearly visible piece of vegetal material between their mandibles. Others deposited fragments of a wet, soft material that they masticated with the pieces of vegetal material, repairing the breaches in 2–3 days, the new carton hardening progressively.

### 3.2 Looking for the presence of fungal mycelia in the carton of the nests

The very hard *C*. *clariventris* carton nests showed a structure formed of numerous relatively small cavities (Figure 2b,c).

**FIGURE 4:**
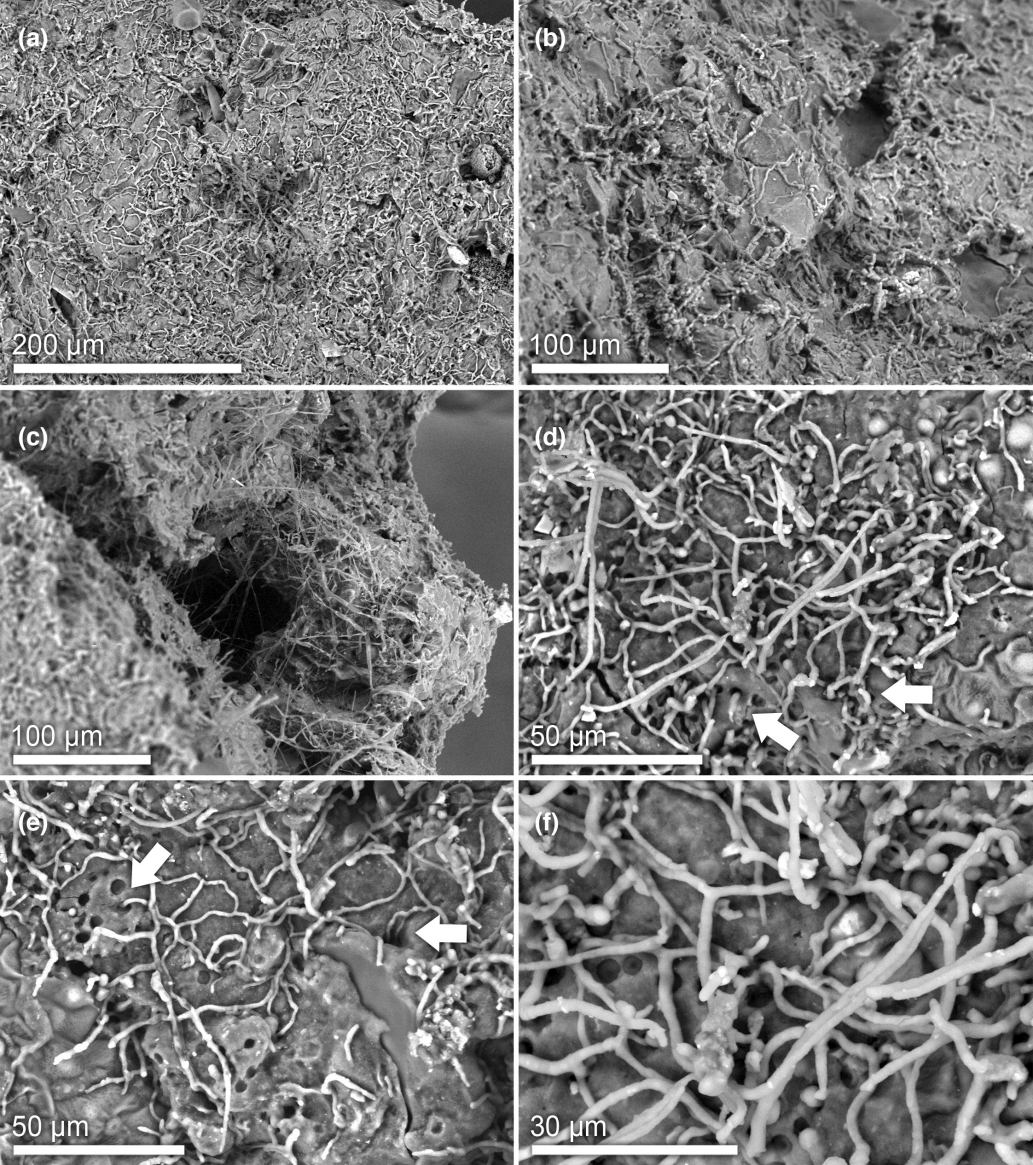
Scanning electron microscope images of the carton of the walls of *Crematogaster clariventris* nests showing the fungal hyphae/mycelium that penetrate the nest carton through pores (arrows).

The observation of *C*. *clariventris* nest walls under scanning electron microscopy revealed the presence of fungal mycelia (Figure 4). The majority of the observed hyphae, 1–3 μm in diameter, were densely branched. A few larger hyphae with clearer cell walls were also observed (Figure 4a). The fungal hyphae were embedded in the fibrous matrix of the nest carton (Figure 4a–c) and ran along the inner wall, forming a network (Figure 4d–f). Numerous hyphae invaded this porous structure (see arrows, Figure 4e,f).

### 3.3 Metabarcoding of nest fungi and screening for known associated fungi

**FIGURE 5:**
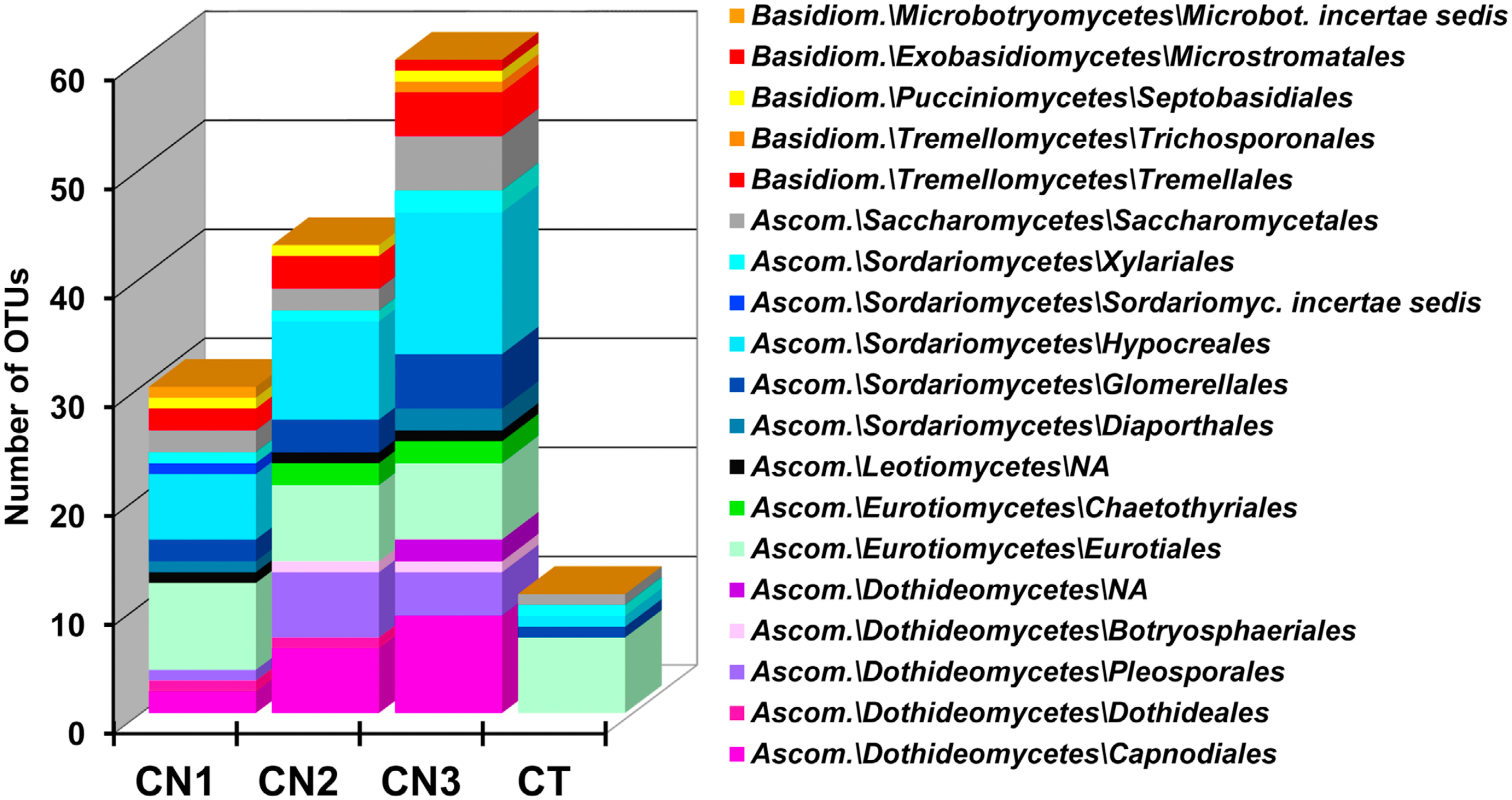
OTU abundance and the distribution of fungal orders for the three nest samples (CN1, CN2 and CN3) and control (CT). A total of 340,790 reads (the average length being 211.5 nucleotides) was obtained after quality filtering. The clustering analysis of these sequences yielded 248 putative OTUs of which we removed those that were unidentifiable and those represented by less than 15 reads. In this way, a final data set of 338,291 reads clustered in 72 OTUs was retained. Fourteen orders of Ascomycota and five orders of Basidiomycota were recorded, the former accounting for 87.5% of the OTUs and 99.73% of the reads (details in Table S1 below). Three orders among the most well represented were also noted in the control: Hypocreales (15 OTUs; 20.8%), Eurotiales (nine OTUs; 12.5%) and Saccharomycetales (five OTUs; 6.9%). The latter two were represented by only one OTU with a high percentage of reads (Table S1 in Dejean et al., 2022). On the contrary, the abundant fungal order Capnodiales (10 OTUs; 13.9%) and the much less represented Chaetothyriales (two OTUs) were noted in the carton of the *C*. *clariventris* nests, while missing from the control sample. The ten other Capnodiales *s*.*l*. and Chaetothyriales OTUs were scattered among different groups of fungal saprobes and pathogens isolated from plants (Figures 6 and 7) and BLAST searches with the ITS1 of these OTUs matched with the sequences of these kinds of fungi (Table 1 below).

The DNA metabarcoding approach used to detect the presence of fungal taxa in intact and repaired parts of *C*. *clariventris* nest walls revealed a very diverse fungal community (248 OTUs; 72 OTUs after eliminating those represented by less than 15 reads). This includes numerous opportunistic and airborne fungi (“contaminants”), as is known for other cases of fungi used in ant constructions (Vasse et al., 2017; Voglmayr et al., 2011). More importantly, 10 OTUs of the fungal order Capnodiales and two OTUs of Chaetothyriales, two orders used in ant constructions, were noted in the nest walls, while missing from the control sample (Figure 5; Table 1).

**TABLE 1:** Blast hits for Capnodiales and Chaetothyriales using GenBank and ant fungi.

Out	No. reads	Best hit accession	E‐value	Similarity (%)	Query cover (%)	Best hit name	Traits/origin of best hit organism	Reference
**Capnodiales**								
**Cre_006041**	4083	**HQ634609**	1.61 × 10 ^−87^	100	100	**Capnodiales sp**. **BN‐Lon33‐1**	**Carton nest of *Crematogaster* sp**.,	Voglmayr et al. ( 2011)
		**HQ634615**	1.61 × 10 ^−87^	100	100	**Capnodiales sp**. **CN‐Cre‐Bo1‐5**	**Cameroon**	
Cre_004151	54	KF901534	1.42 × 10 ^−82^	100	87	Unknown *Neodevriesiaceae*	From leaf disease of *Corymbia henryi*, Australia	Quaedvlieg et al. ( 2014)
Cre_083691	51	KX287269	6.57 × 10 ^−86^	99.4	100	*Acrodontium crateriforme* strain CBS 842.71	Saprobic from leaf of *Citrus* sp., Indonesia	Videira et al. ( 2016)
Cre_062921	48	GU214635	2.16 × 10 ^−64^	91.8	100	*Devriesia strelitziicola* strain ×1045	From leaves of *Strelitzia* sp., South Africa	Crous et al. ( 2009)
Cre_059371	48	GU068516	6.37 × 10 ^−25^	90.4	52	*Teratosphaeria australiensis* culture‐collection MUCC:731	From leaves of *Corymbia ficifolia*	Taylor et al. ( 2015)
Cre_221461	42	MF965983	3.10 × 10 ^−47^	86.4	100	Fungal sp. clone ITS1_OTU_2120	–	Sutcliffe et al. (2018)
Cre_123451	41	MK442573	1.83 × 10 ^−86^	100	100	*Cercospora gomphrenigena* culture CBS 144613	From *Gomphrena globosa* (*Amaranthaceae*), South Africa	Crous et al. (2019)
Cre_248051	34	GU214635	8.61 × 10 ^−48^	88.6	90	*Devriesia strelitziicola* strain ×1045	From leaves of *Strelitzia* sp.	Crous et al. (2009)
Cre_004821	27	AJ307020	9.36 × 10 ^−67^	94.2	96	*Phloeospora mimosae‐pigrae* isolate Cuba	Pathogen on *Mimosa pigra*, Cuba	Hennecke et al. ( 2001)
Cre_466851	15	JN232423	6.84 × 10 ^−43^	85.7	88	*Teratosphaeria pseudafricana* strain PM16	Leaf diseases on *Eucalyptus* *globulus*, Brazil	Teodoro et al. (2012)
**Chaetothyriales**								
Cre_055281	42	KU204526	7.25 × 10 ^−114^	99.6	100	*Cyphellophora guyanensis* voucher INBio:167A	From *Platymiscium curuense* (Fabaceae), Costa Rica	K. Rojas‐Jimenez, C. Murillo, J. Clardy, unpublished
**Cre_320021**	19	**KX822548**	7.45 × 10 ^−48^	82.1	100	**Chaetothyriales sp**. **PF51b**	**Rubiaceae domatia with *Crematogaster margaritae***,	Voglmayr et al. ( 2011)
		**KX822549**	7.45 × 10 ^−48^	82.1	100	**Chaetothyriales sp**. **PF52c strain CBS 134918**	**Cameroon**	Vasse et al. ( 2017)

Note: Bold indicates accessions corresponding to ant nests.

**FIGURE 6:**
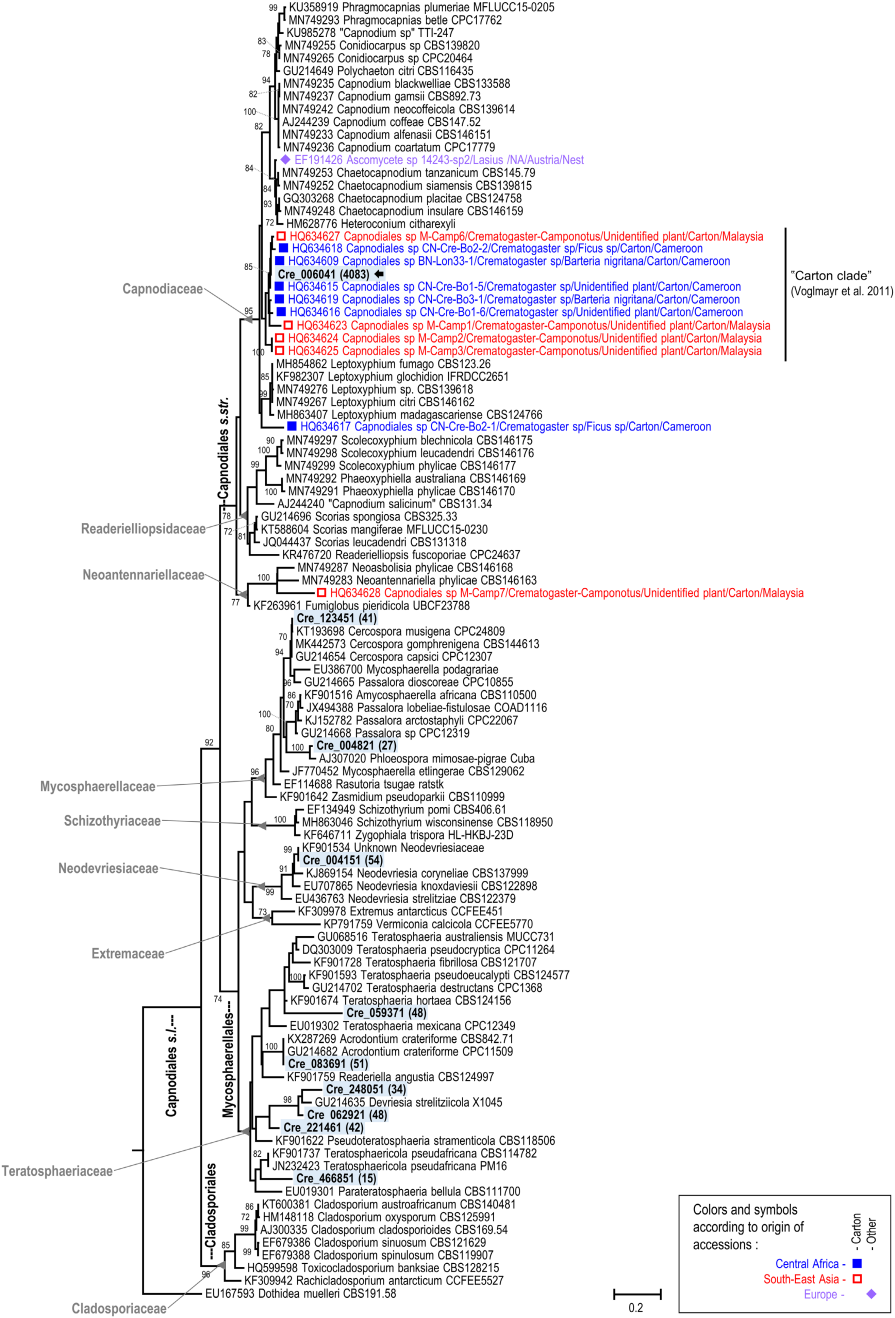
Maximum likelihood tree based on ribosomal ITS sequence alignment for Capnodiales *s*.*l*. including some known ant‐associated fungi. Bootstrap values above 70% are presented. This tree shows the phylogenetic positions of 10 OTUs recorded through metabarcoding (blue shaded labels in bold with number of reads between parentheses), particularly OTU Cre_006041 (black arrows). Colors correspond to ant‐associated fungi according to their geographical origin. Squares indicate isolates from ant‐made carton and diamonds from other material.

Among these 12 fungal OTUs, the OTU Cre_006041 of the Capnodiales is represented by a high number of reads in the metabarcoding dataset (4083 reads, see Table S1 in Dejean et al., 2022). On the constructed Capnodiales phylogeny, this OTU appears in a clade of fungi known for their relationships with ants (see “Carton Clade 3” in Figure 6). Indeed, its ITS1 sequence is identical to those (accessions HQ634609 and HQ634615) of two fungal isolates (BN‐Lon33‐1 and CN‐Cre‐Bo1‐5) found in the carton of the nests of an unidentified Cameroonian (African) *Crematogaster* (Voglmayr et al., 2011) (100% similarity; Table 1). Thus, the abundance and the nature of the OTU Cre_006041 of the Capnodiales suggest that the structural function noted in Figure 4 is mostly due to the mycelium of this OTU.

**FIGURE 7:**
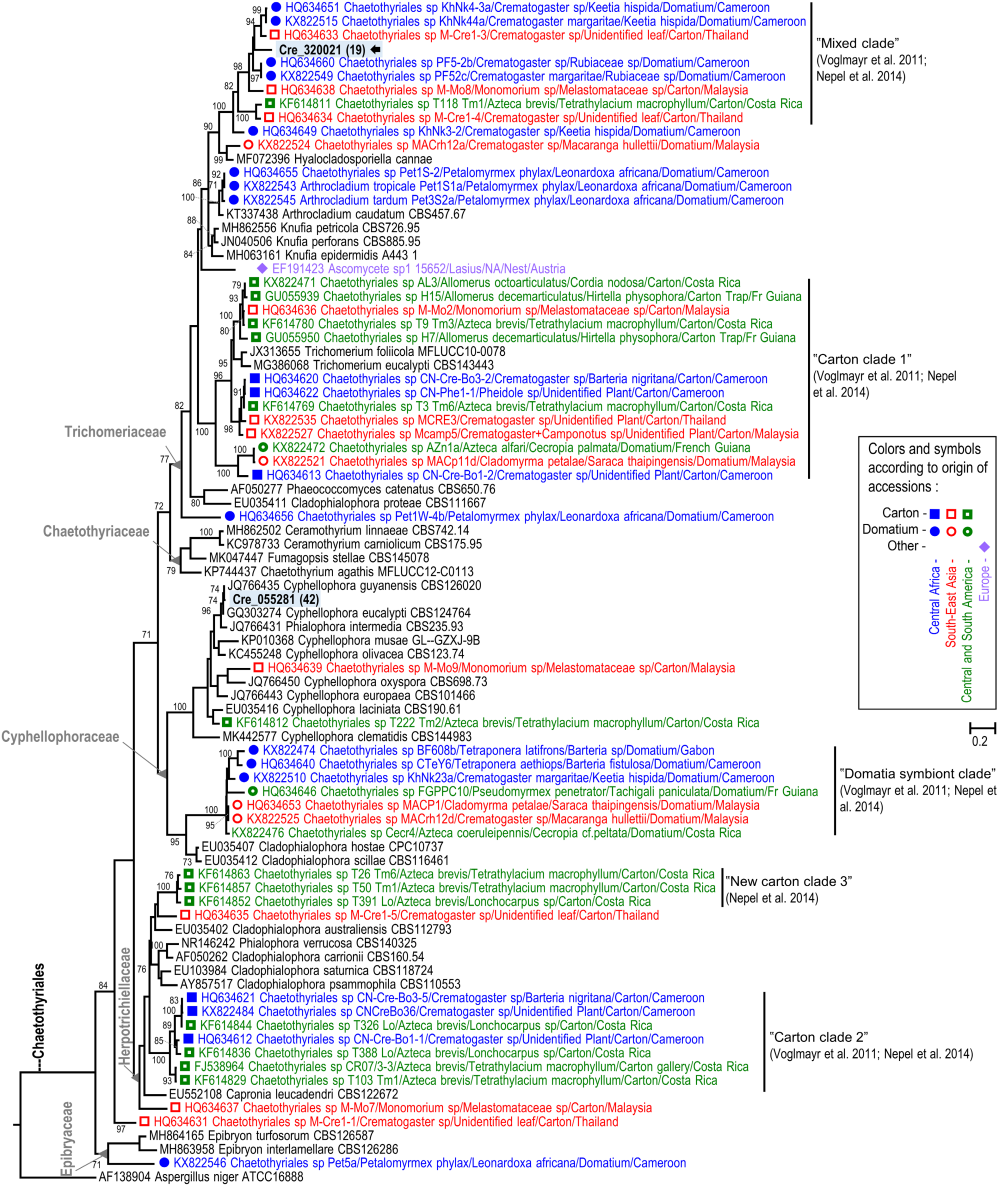
Maximum likelihood tree based on ribosomal ITS sequence alignment for Chaetothyriales including some known ant‐associated fungi. Bootstrap values above 70% are presented. This tree shows the phylogenetic positions of the two OTUs recorded through metabarcoding (blue shaded labels in bold with number of reads between parentheses), particularly OTU Cre_320021 (black arrows). Colors correspond to ant‐associated fungi according to their geographical origin. Squares indicate isolates from ant‐made carton, circles from domatia and diamonds from other material.

Also, a phylogenetic analysis placed the OTU Cre_320021 of the Chaetothyriales in a clade of ant‐associated fungi (Nepel et al., 2014; Voglmayr et al., 2011) (see “Mixed Clade” in Figure 7). Indeed, a BLAST search with ITS1 for OTU Cre_320021 found as best hits, but with low similarity (82.1%), two accessions (KX822548 and KX822549; Table 1) of two isolates (PF51b and PF52c CBS 134918) associated with the African plant‐ant species *C*. *margaritae* (Vasse et al., 2017; Voglmayr et al., 2011). However, OTU Cre_320021 was recorded in only two of the three tested nest samples and at a very low level (1 and 18 reads recorded in CN2 and CN3 respectively; see Table S1 in Dejean et al., 2022).

In the constructed ITS phylogenies, the nine other Capnodiales OTUs and the other Chaetothyriales OTUs are found outside the clades of ant‐associated fungi (Figures 6 and 7), and they mainly correspond to saprobes or pathogens of plant leaves (Table 1).

### 3.4 Amplification of rDNA ITS of OTU Cre_006041 from nests and ants

**FIGURE 8:**
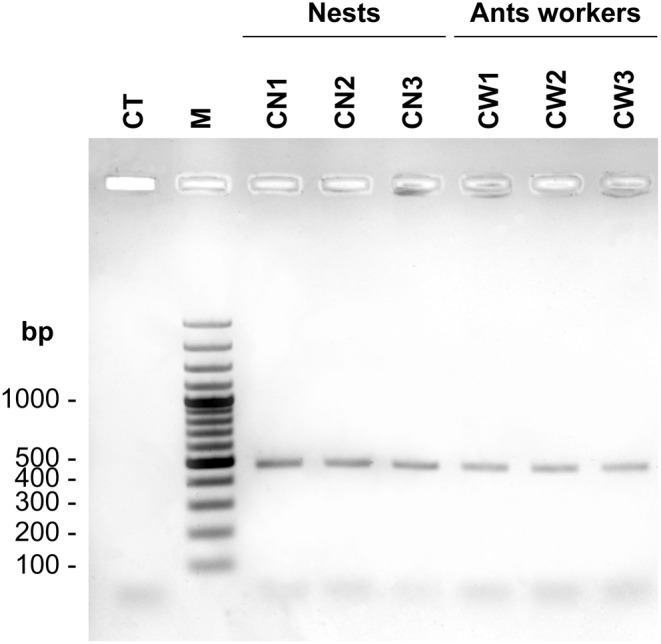
Detection of the Capnodiale OTU Cre_006041 in DNA extracted from three nest samples (CN1, CN2 and CN3) and from ant workers from these nests (CW1, CW2 and CW3) by PCR‐amplification of rDNA ITS with CreCapnoF specific primer and ITS4 universal primer. CT is the control sample. M is the 100pb Plus DNA ladder (Thermofischer) used as molecular weight marker. A same‐sized fragment is amplified in all nest and worker samples. Subsequent sequencing of PCR products shows that the nest fragments, and the workers tested share the same fragment whose sequence matches with the ITS1 sequence of OTU Cre_006041.

PCR amplifications performed with CreCapnoF/ITS4 primers yielded an identical PCR product of *c*.*a*. 485 pb for the three nest samples, and the workers' bodies analyzed (Figure 8). Sequences of these PCR products are identical, and they match with the ITS1 sequence of the OTU Cre_006041 and with the ITS1‐5.8S‐ITS2 sequence of the CN‐Cre‐Bo1‐5 fungal isolate from the Cameroonian species *Crematogaster* sp. (accession HQ634615; Voglmayr et al., 2011).

## 4 DISCUSSION

Noting that workers retrieving pieces of vegetal material cooperate with others transporting a soft compound to repair damaged nests, with the resulting composite material containing fibers from the Capnodiales, permitted us to deduce that *C*. *clariventris* is a defoliator using vegetal material to grow fungi. Thus, leaf‐cutting, fungus‐growing behaviors are not restricted to higher leaf‐cutting Attina of the genera *Atta*, *Acromyrmex* and *Amoimyrmex*. This similarity between taxa belonging to two distinct ant tribes of the subfamily Myrmicinae (Crematogastrini vs. Attini) and originating from two distinct geographical areas (Africa vs. the New World) represents an example of convergent evolution.

However, whereas the Attina cultivate Basidiomycota, mostly Agaricaceae, for food (Branstetter et al., 2017), *C*. *clariventris* workers grow Ascomycota Capnodiales to reinforce the carton of their arboreal nests. Thus, this does not correspond to “fungal cultivation” or, more exactly, to “true fungal agriculture” based on the following traits: (1) planting, (2) cultivation and (3) harvesting the crop for food in an obligate dependency (Mueller et al., 2005; see also Campbell et al., 2023). Three plant‐ants of the families Formicinae and Pseudomyrmecinae feed on Chaetothyriales that line their domatia, but there is not obligate dependence as they also tend hemipterans (Blatrix et al., 2012). In addition, plant cultivation by ants was noted for ant gardens (i.e., mutualistic associations between certain arboreal ants and various epiphytes) and Fijian agriculture involving *Philidris nagasau* (Dolichoderinae) that obligatorily cultivate *Squamelaria* epiphytic myrmecophytes (Rubiaceae). Here, although in certain cases the ants obtain food through extra‐floral nectar, the mutualism is based on epiphyte seedlings being provided nutritional resources in exchange for a nesting place (i.e., farming for shelter) (Campbell et al., 2023). For ants building their nests using a fungal mycelium, including *C*. *clariventris*, the mycelium is likely introduced by them (i.e., a kind of specificity) and “fertilized” via the pulp of malaxated young pieces of plants (this study). This permits the ants to build their shelter (the carton nests), but, after the death of the mycelium, the hyphae's cell walls remain sturdy, forming a natural composite material (Voglmayr et al., 2011; for the use of mycelium in material construction see Jones et al., 2020; Yang et al., 2021).

Furthermore, the mode of leaf cutting differs. While leaf‐cutting Attina workers use their mandibles asymmetrically in an elaborate behavior permitting them to rapidly cut comparatively large leaf pieces (Schofield et al., 2011; Schultz, 2021), *C*. *clariventris* workers cut small pieces with a typical chewing motion of their mandibles moving symmetrically (Paul, 2001; this study).

All Attina, plus some plant‐ants and *Lasius* species that reinforce their nests with fungal hyphae transmit their fungi vertically, although horizontal transmission between species also exists (Mayer et al., 2018; Phillips et al., 2021; Poulsen et al., 2009; Schlick‐Steiner et al., 2008). Yet, we were never able to capture *C*. *clariventris* swarming queens because the nests are too high on canopy trees (Dejean et al., 2016) and because swarming is likely diurnal, preventing us from capturing queens using light traps.

However, that the source of the fungi involved in reinforcing *C*. *clariventris* nests has not been shown in no way detracts from the fact that this ant species has evolved relationships with fungi. Indeed, we noted the presence of the OTU Cre_006041 of the Capnodiales that matches the fungal isolates found in the hard carton of the nests of the Cameroonian *Crematogaster* sp. (Voglmayr et al., 2011). This OTU was noted in both the carton of *C*. *clariventris* nests and in the workers' bodies showing that they likely manipulate this fungus; indeed, the hyphae can be carried in the infrabuccal pocket or on the gaster (Pringle & Moreau, 2017).

This was not the case for the OTU Cre_320021 of the Chaetothyriales that, noted in two out of three nests and at a very low level, may come from plant leaves and attended hemipterans (Pringle & Moreau, 2017). Belonging to a “mixed clade” containing both domatial fungal symbionts and carton fungi, it is known to be associated with the plant‐ant *C*. *margaritae* and the myrmecophyte *Keetia hispida* (Rubiaceae) in Cameroon (Vasse et al., 2017; Voglmayr et al., 2011), so that its role in reinforcing the nests is unlikely or very low.

For some *Crematogaster* species, both hard carton nests and defoliation were noted, but the correlation between them was not shown. On the one hand, the nests of African *Crematogaster* from the subgenus *Atopogyne* are reinforced by Capnodiales indicating a special association characterized by extremely hard carton nests with dense textures (Taylor, 2015; Voglmayr et al., 2011). Other carton‐nesting ants, including other *Crematogaster*, use Chaetothyriales to reinforce their carton, which is brittle (Quan et al., 2021, 2022; Voglmayr et al., 2011). On the other hand, defoliating activities have been observed in two African *Atopogyne*, *C*. *buchneri* and *C*. *africana*, whose workers attack the leaves, buds and flowers of several plants (Eguagie, 1973; Taylor, 2015). This was also noted for *C*. *wellmani*, another African species of the subgenus *Sphaerocrema* (Bruno de Miré, 1969).

## 5 CONCLUSION

Leaf‐cutting, fungus‐growing abilities are not specific to the higher Attina, a subtribe limited to the New World, but are also found in a *Crematogaster* species from the Old World. However, fungal mycelia, rather than being cultivated for food, are in this case used to reinforce the carton of the nests through the formation of a hard composite material that resists heavy equatorial rains.

## AUTHOR CONTRIBUTIONS

**Alain Dejean:** Data curation (equal); formal analysis (equal); writing – original draft (equal). **Piotr Naskrecki:** Investigation (equal). **Christian Faucher:** Formal analysis (equal); methodology (equal). **Frédéric Azémar:** Formal analysis (equal); investigation (equal); methodology (equal); writing – review and editing (equal). **Maurice Tindo:** Conceptualization (equal); investigation (equal); methodology (equal). **Sophie Manzi:** Formal analysis (equal). **Hervé Gryta:** Conceptualization (equal); formal analysis (equal); methodology (equal); writing – original draft (equal).

## CONFLICT OF INTEREST STATEMENT

The authors declare no conflicts of interest.

## Data Availability

Two tables and the datasets generated and/or analyzed during the current study are available in the Figshare Data Repository doi:10.6084/m9.figshare.14452692.v5 (Dejean et al., 2022).
